# Multi-omics research strategies in traditional Chinese medicine: A review

**DOI:** 10.1097/MD.0000000000045479

**Published:** 2025-10-31

**Authors:** Chenqiong Xie, Yiming Che, Yanxia Zhou, Jinjin Wu, Jinglin Shen

**Affiliations:** a Department of Traditional Chinese Medicine Pharmacy, The Third Affiliated Hospital of Zhejiang Chinese Medical University, Hangzhou, Zhejiang, China; b Department of Acupuncture, Tuina and Rehabilitation, Shandong College of Traditional Chinese Medicine, Yantai, Shandong, China; c Department of Pharmacy, Jiangxi Medical College, Shangrao, Jiangxi, China.

**Keywords:** authentication, multi-omics, processing mechanisms, quality control, safety evaluation, traditional Chinese medicine

## Abstract

Traditional Chinese medicine (TCM) has been employed to treat a wide range of ailments for millennia. Although the active ingredients of TCM typically have lower concentration of active compounds compared to Western medications based on conventional metrics, they frequently exert synergistic therapeutic effects on multiple targets. These varied effects hinder widespread adoption by making it challenging to evaluate efficacy and comprehend underlying mechanisms. High-throughput omics methods, particularly genomics, proteomics, metabolomics, and transcriptomics, open up new avenues for discovering biomarkers related to quality control, safety evaluation, authentication, and elucidating processing mechanisms. This paper reviews developments in these omics over recent decades, summarizes their applications in pharmaceutical research for quality control, toxicity evaluation, processing, and authentication, and discusses omics limitations. We also highlight how integrative omics and systems biology can advance TCM understanding and standardization.

## 1. Introduction

The application of traditional Chinese medicine (TCM) in contemporary healthcare is expanding globally. The TCM sector has achieved significant progress in pursuing modernization over the past 2 decades.^[1,2]^ However, innovation and preservation of TCM traditions remain challenges for both academia and industry. The modernization and global integration of TCM are hindered by the lack of standardized quality control protocols. Unlike synthesized pharmaceuticals, TCM preparations face greater difficulties in quality assurance due to their complex multicomponent chemistry and diverse preparation methods. Key factors affecting TCM quality include genetic variations, cultivation practices, environmental conditions, harvest timing and processing techniques.^[3]^ Therefore, establishing scientifically rigorous quality assessment systems that address the entire production chain while respecting TCM principles is essential to ensure safety and therapeutic efficacy.

Omics refers to the collective characterization and quantification of molecules that constitute a cell, tissue, or organ. It includes metabolomics, transcriptomics, proteomics and genomics, along with other omics subjects such as phosphoproteomics, peptidomics or lipidomics. By interpreting the intricate web of pathways and providing a comprehensive understanding of the processes under study, omics techniques provide a deep picture of the multilayer functional molecular landscape.^[4]^ Metabolomics has been effectively used in the analysis of TCM studies. It utilizes strong analytical tools, such as MS-hyphenated separation techniques and bioinformatics, to quantify the metabolites generated or changed in biological organisms. This approach has shown significant promise for clarifying the treatment mechanisms behind TCM and for advancing our knowledge of metabolic pathways and disease-associated biomarkers more broadly.^[5]^ Genomics is the study of genomes (including human genomes and therapeutic organisms) and their interactions. It utilizes technologies such as genome sequencing and bioinformatics algorithms to decode genetic information related to TCM, uncover regulatory networks, and elucidate the molecular functions of bioactive components.^[6]^ By utilizing sophisticated proteomics technologies, such as 2-dimensional gel electrophoresis, multi-dimensional liquid chromatography, matrix-assisted laser desorption/ionization (MALDI)-time of flight mass spectrometry (MS), and isobaric tags for relative and absolute quantitation, proteomics enables comprehensive protein studies. This facilitates drug target discovery, bioactive component identification, and mechanistic clarification of therapeutic actions.^[7]^ Transcriptomics, as a powerful tool for elucidating disease mechanisms and identifying biomarkers, not only anhances clinical practice by improving diagnostic precision, but also enables researchers to assess alternative splicing and polyadenylation events, identify fusion transcripts, explore noncoding RNAs and refine transcript annotation, and discover novel transcripts.^[8,9]^ Multi-omics technologies have provided in-depth insights into biomolecules and cellular constituents, including RNAs, proteins, metabolites, and genomic elements. Integrative approaches such as transcriptomics, proteomics, metabolomics, and whole-genome sequencing are generating promising results, with enhanced synergies when applied to the same patient cohort. This convergence enables comprehensive molecular profiling of TCM, facilitating development of targeted therapies, prediction of treatment responses, optimization of diagnostic and therapeutic strategies and scientific validation of TCM safety and efficacy.

This report systematically reviews research activities from 2014 to 2024 in TCM, focusing on medicinal materials and compound prescriptions. Analysis reveals that omics technologies have been employed to haracterize chemical compositions and their batch-to-batch variations, assess pharmacological activity and toxicological profiles, elucidate processing mechanisms (Paozhi), and authenticate herbal materials to combat adulteoverall process quality controlaches enable time-resolved molecular profiling and pathway analysis. These findings provide a scientific foundation for advancing TCM standardization and overall process quality control.

## 2. The development of TCM

TCM, with its 3 millennia of clinical application in China, is recognized as a highly structured system of herbal medicine globally.^[10]^ Classification and effectiveness principles of TCM are documented in classic texts including Shen Nong’s Materia Medica and the Basic Compendium of Materia Medica.^[11]^ The effectiveness and safety profiles of TCM have been established through millennia of empirical experience in its use. TCM continues to play a significant role in healthcare as complementary and alternative medicine, for disease prevention, treatment, and as a therapeutic approach. It is applied in managing conditions such as cardiovascular diseases,^[12]^ cancer,^[13]^ cerebrovascular diseases,^[14]^ inflammatory disease,^[15]^ coronavirus disease (Covid-19).^[16,17]^ Despite extensive research from preclinical (animal, cell culture) to clinical (epidemiological, trial) studies supporting TCM’s disease prevention and treatment potential, significant translational challenges persist. Standardized quality control protocols must be established to ensure reproducibility, particularly regarding compatibility assessments of active compounds within multi-herb formulations. Concurrently, systematic evaluation of potential drug-herb interactions and cross-reactivities is essential for safe integration with conventional therapies like chemotherapy. As TCM gains global traction, rigorous validation of its efficacy and safety profiles through advanced toxicological profiling-distinguishing therapeutic versus harmful constituents-becomes increasingly critical.^[18]^ Furthermore, identifying molecular biomarkers is pivotal for authenticating TCM components, elucidating mechanisms of action, and objectively evaluating therapeutic outcomes.^[19]^ Modern multi-omics approaches have begun identifying relevant chemical indicators, providing foundational tools to address these challenges and advance TCM toward evidence-based clinical practice.

## 3. The current situation of multi-omics approaches

In contrast to traditional research methods characterized by high time investment, cost, and risk, omics technologies offer superior sensitivity and resolution. These advanced methodologies provide comprehensive solutions for critical pharmaceutical challenges, including drug quality/safety evaluation, mechanism elucidation, resource authentication, and novel compound discovery. Historically, omics has evolved as a transformative molecular tool, becoming indispensable in modern pharmacological and biological research. A systems-level comprehension of cellular biomolecules-encompassing genomic, proteomic, and metabolomic constituents-is fundamentally dependent on omics-based analytical platforms.

The genetic code serves as the universal foundation of biological systems. Consequently, understanding core DNA sequences and gene encoding mechanisms has become essential to modern biology.^[20]^ Recent years have witnessed transformative advances in plant genome sequencing methodologies, marked by key milestones: pre-2012 plant genome releases,^[21]^ angiosperm genomes assemblies before 2018,^[22]^ third-generation sequencing’s impact on plant genome construction,^[23]^ the Herb Genome Program^[24]^ and the 1000 Medicinal Plants Genome Project.^[25]^ Single-cell sequencing now enables detection of genomic structural variations and single-nucleotide polymorphisms at cellular resolution.^[26]^ However, limitations persist due to the lack of standardized protocols for whole-genome amplification and unified data annotation frameworks. Integrating multi-platform datasets through advanced bioinformatic approaches significantly enhances data reliability and biological insights.

Transcriptomics study reveals the profiles of RNA species, such as mRNA, rRNA, tRNA, and other noncoding RNAs, that are created in a cell. It shows how gene expression varies depending on the situation.^[27]^ The transcriptome is akin to an individual biological fingerprint, in which gene activity serves as an indicator of cell function, developmental stage, or aberrations caused by both internal and external stressors. This idea has fascinating ramifications because, in theory, a single-cell’s transcriptome would contain all the data required to identify, trace, and reconstruct the cell’s ontogeny within a complex multicellular tissue, organ, or even the entire organism. These days, single-cell and spatial transcriptomics are crucial to TCM research and can help address TCM’s compositional heterogeneity. For instance, researchers have found that, at the organ level, stress defense genes are upregulated while growth-related, energy-consuming activities are downregulated when using standard transcriptomics to assess drought-induced transcriptome alterations. Notably, single-cell transcriptome data revealed that while the majority of genes were elevated in the epidermis, the mesophyll displayed a considerable downregulation of genes under mild drought stress. Understanding the intricate response of plants to environmental stimuli requires deciphering single-cell networks. Single-cell transcriptomics is a powerful tool for identifying tissue-specific stress-responsive pathways, enabling selective modification in target tissues without inducing unanticipated side effects in other tissues or organs.^[28]^ However, these techniques exhibit inherent drawbacks, such as limited applicability and spatial resolution. Subsequently, integrated approaches have been proposed, including cross-species root single-cell analysis and multi-omics integration to surpass the limitations of individual omics analyses.^[29]^

Proteomics is a vital source of knowledge about biological systems because it is based on the enormous analytical power to characterize identification, quantification, interactions, functions, and enzymatic activities of proteins/peptide, which are the primary structural and functional determinants of cells. The explosive rise of proteomics offers a plethora of new techniques for integrating TCM with contemporary technology and systems biology, potentially propelling TCM’s modernization and internationalization forward.^[30]^ Specifically, systems biology techniques like comparative proteomics and proteome profiling, which enable system-wide measurement and quantification of treatment responses at the proteome level, can evaluate the therapeutic benefits and side effects of TCM remedies. MS serves as the cornerstone of proteomics analysis for high-throughput protein/peptide identification. In TCM research, multiple techniques have been synergistically applied, including MS-coupled computational algorithms, instrument control language, MALDI, electrospray ionization, and complementary chromatographic methods. These approaches have advanced investigations of TCM’s pharmacological mechanisms, herb authentication, and acupuncture-induced protein expression profiles in animal models.^[31]^

Metabolomics enables both qualitative and quantitative analysis of the multifaceted, dynamic metabolic responses to pathological and physiological stimuli in living systems.^[32]^ Nuclear magnetic resonance (NMR) and MS serve as the predominant downstream techniques for metabolite identification. Current metabolic research spans diverse scales, from population studies to organelle-level investigations, with novel technologies facilitating multilevel analyses. Notably, 3 key approaches have emerged, functional screening of cellular responses to metabolic stress, small-scale metabolomics for organelles and rare cell types, and noninvasive imaging for evaluating disease-related metabolic disturbances.^[33]^ Advances in single-cell analytical methods now allow systematic examination of cellular phenotypes through integrated transcriptomic, proteomic, metabolomic, and genomic profiling.^[34]^ These technological and analytical breakthroughs have enabled comprehensive cellular metabolism analysis, which has accelerated the discovery of novel metabolic pathways.^[35]^

Principal component analysis and partial least squares represent 2 widely used multivariate statistical methods for pattern recognition in NMR and MS datasets.^[36]^ Additionally, major computational platforms for metabolomics analysis comprise XCMS,^[37]^ MZmine,^[38]^ MetAlign,^[39]^ MathDAMP,^[40]^ and MALDI.^[41]^

## 4. The application of multi-omics in TCM

### 4.1. Quality control

TCM has been attracting increasing global attention due to its unique properties in diseases prevention and treatment. Although TCM has a long history of clinical application, standardized quality control remains a key challenge for its modernization. Specifically, TCM quality can be influenced by multiple factors, including plant species, geographical origin, harvest period, plant parts, and storage conditions. For mechanistic studies of therapeutic effects, novel assessment systems should be developed, and more rigorous scientific quality control protocols need to be implemented.

The multi-origin (multisource) property of TCM may lead to quality control challenges. Zhang et al^[42]^ developed an integrated approach combining chemometrics analysis with UHPLC/Q-Orbitrap-MS to differentiate 4 Pulsatilla Adans species. Oleanane- and lupane-type triterpenoid saponins were distinguished by characteristic fragment ions at *m*/*z* 347 and their fragmentation patterns. Using a non-targeted bidirectional screening method, 157 triterpenoid saponins were identified, including 34 previously unreported constituents in PSA herbs. The study identified 12 chemical markers suitable for PSA quality control.

Li et al^[43]^ employed LC-MS to examine metabolome variance within Mu Dan Pi derived from 372 tree peony cultivars. Using this method, Tao et al^[44]^ identified 6 components (ent-18-acetoxy-17-hydroxy-16βH-kauran-19-oic acid and ent-18-acetoxy-16α, 17-dihydroxykauran-19-oic acid, 7,3′, 4′-trimethoxy luteolin and (4β)-19-hydroxy-kaur-16-en-18-oic acid, (6S,7E)-6,9-dihydroxymegastigma-4,7-dien-3-one and quercetin) as promising chemical markers to differentiate *Siegesbeckia pubescens makino, Sigesbeckia orientalis* L., and *Sigesbeckia glabrescens makino*, respectively. Furthermore, studies have identified betaine, Krebs cycle intermediates, phenylethanoid glycosides, and iridoids as molecular markers for distinguishing chemical profiles between *Cistanche deserticola* and *Cistanche tubulosa*,^[45]^ used acetylated gingerol derivatives as the ginger indication instead of gingerol or shogaol,^[46]^ conducted research on structural characterization and discrimination of *Clematidis radix et rhizoma* with multiple botanical origins,^[47]^ identified flavonoids as quality markers for *Sophora flavescens*,^[48]^ and elucidated the formation mechanism of *Stiff silkworm. Bombyx batryticatus*,^[49]^ all successfully performed using LC-MS.

Compounds with low molecular weights, low polarity, low boiling points, or volatility are suitable for GC-MS analysis. GC-MS was used to compare the composition and antioxidant activities of volatile oils from Mahuang (MH, the stems of *Ephedra sinica*) and Mahuanggen (MHG, the roots of *E sinica*). Based on the projection value of orthogonal partial least squares discriminant analysis and the *P* value of the Mann–Whitney test, 32 differential chemical markers were identified. Additionally, most MH samples contained significantly higher concentrations of 2 pharmacologically significant molecular marker – α-terpineol – compared to MHG samples. Antioxidant assays demonstrated that MH exhibited significantly stronger free radical scavenging activity than MHG.^[50]^

Despite its relatively lower sensitivity, NMR can detect a wide range of compounds in TCMs. Li et al^[51]^ investigated the antitussive and expectorant properties of Farfarae Flos collected at 2 developmental stages (flower buds and fully opened flowers). NMR-based metabolomic analysis revealed potential correlations between specific chemical components (4,5-*O*-dicaffeoylquinic acid, caffeic acid, chlorogenic acid, 3,5-*O*-dicaffeoylquinic acid, 3,4-*O*-dicaffeoylquinic acid, rutin, kaempferol analogues, 2,2-dimethyl-6-acetylchromanone, EMDNT, tussilagone, baueren-7-ene-3β, 16α-diol, β-sitosterol, sitosterone) and these pharmacological effects. However, the primary active components responsible for Farfarae Flos’s expectorant and antitussive properties were identified as endogenous metabolites. By applying ^1^H-NMR spectroscopy, Wang et al^[52]^ comprehensively characterized the compositional differences between Chinese warm temperate propolis and Chinese mid-temperate propolis. Similarly, Hua et al^[53]^ successfully identified 14 significantly differentiated metabolites in *Pseudostellariae* radix from different cultivation sites, using an integrated approach of ^1^H-NMR and HPLC.

In an effort to fully utilize the advantages of diverse approaches, researchers have recently attempted to combine multiple analytical methodologies. By integrating LC-MS, DNA barcoding, and electronic nose techniques, 4 distinct cultivars of Citri Reticulatae Pericarpium cultivars were identified. Li et al^[54]^ applied LC-MS combined with DNA barcoding to analyze Qinjiao from 4 species (*Gentiana macrophylla* Pall*., Gentiana straminea* Maxim*., Gentiana crassicaulis Duthie ex* Burk. and *Gentiana dahurica* Fisch.), conducting a comprehensive quality evaluation. The aforementioned studies suggest that systematic analytical methodologies are essential to achieve comprehensive and accurate quality control for TCMs.

Additionally, recent applications of omics technologies for TCM quality control are systematically summarized in Table 1. Table 1 demonstrated that ^1^H-NMR and MS (GC-MS and LC-MS) are the primary analytical methods used for TCM samples, with LC-MS particularly effective for analyzing high molecular weight, thermally unstable, and high boiling point chemicals.^[48–72]^

**Table 1:** Omics technology studies on quality control of TCHs.

Factor	Sample	Method	Description	References
Geographical	Chinese warm temperate propolis (CWTP) and Chinese mid-temperate propolis (CMTP)	^1^H-NMR	Chrysin, apigenin, galangin,pinocembrin, kaempferol, 3, 4-dimethylcaffeic acid, caffeic acid, and isoferulic acid are consider active compounds	^[48]^
Harvest time	*B batryticatus* with a stiff time of 1, 5, and 9 d (D1, D5 and D9, respectively)	UPLC-MS	Quercetin-7-*O*-β-d-4-*O*-methylglucoside was promised to be used as an index component of quality control	^[49]^
Harvest time	Fully opened flower and flower buds of Farfarae Flos (FF)	NMR	The flower buds differed greatly from the fully opened flower both on the secondary and primary metabolites	^[51]^
Species	*Cistanche deserticola* (CD) and *C tubulosa* (CT)	HPLC-MS	A total of 379 metabolites were annotated out of the 513 that were found and roughly quantified. Betaine, phenylethanoid glycosides, iridoids, and Krebs cycle intermediates were identified as the molecular markers in charge of differentiating the chemical profiles between CD and CT	^[56]^
Species	“Shokyo” (dried rhizome of *Z officinale var. rubens*), “Kankyo” (steamed and dried rhizome of *Z officinale var. rubens*) from Kampo (traditional Japanese) medicine, “Red ginger” (rhizome of *Z officinale var. rubra*) from Indonesian traditional medicine (Jamu), “Shoga” (fresh rhizome of *Z officinale var. rubens*) from Japan and “White ginger” (rhizome of *Z offic inale var. amarum*) from Indonesia	HPLC-MS	Gingerol derivatives that have been acetylated rather than gingerol or shogaol as the pharmacological indication	^[57]^
Species	372 tree peony cultivars	UHPLC-MS	The most prevalent phytochemicals are 42 metabolites, which include oxypaeoniflorin (peak 4), apiopaeonoside, paeonolide, paeonoside, paeoniflorin, PGG, galloylox ypaeoniflorin, and paeonol. As for the extraction and isolation of these useful chemicals, paeonol, paeoniflorin, PGG, methyl gallate, and gallic acid should also be taken into consideration	^[58]^
Species	*Ephedra sinica, E intermedia*, and *E equisetina*	DI-MS	Important MS features in this intraspecies variation were identified as 5 possible target metabolites: methylphenephrine, ephedrine, or pseudoephedrine, and norrephedrine	^[59]^
Species	Forty-two batches of Rhodiola market samples (i.e., not authenticated)	^1^H-NMR, HPTLC	Identified 5 distinct Rhodiola species and offers potential techniques for measuring various species within mixes	^[60]^
Species and harvest time	*Citrus reticulate Blanco, Citrus aurantium* cv Xiucheng, *Citrus aurantium* L., *Citrus aurantium* cv Jizicheng, *Citrus aurantium* xP. trifoliata, *Citrus aurantium* cv Daidai, *Citrus aurantium* cv Morocco sour orange	UPLC-MS	3 of the metabolic pathways were considered to be the most pertinent to the ripening stages in both the QP/CP and ZS/ZQ groups, including flavone and flavonol biosynthesis, flavonoid biosynthesis and phenylpropanoid biosynthesis	^[61]^
Species	*Clematis manshurica* Rupr.*, C chinensis osbeck. and C hexapetala pall.*	UPLC-MS	49 triterpenoid saponins, The PDVs and corresponding marker compounds were identified	^[62]^
Species	*Curcumae wenyujin, C kwangsiensis, C phaeocaulis* and *C longa*.	UHPLC-MS	Curcumae Radix (Yujin) can be identified using curcumin, curcumenone, curcumenol, and zederone as distinct chemical markers.	^[63]^
Geographical	Alisma orientale (Sam.) Juzep (Alismaceae) form Sichuan (SC), Fujian (FJ), and Jiangxi (JX) Provinces	UHPLC/Q-TOF-MS	23 potential triterpene markers contributed mostly to the observed chemical differences	^[66]^
Geographical	*Ophiopogon japonicus* (Thunb.) Ker-Gawl from Zhejiang and Sichuang	UPLC/Q-TOF MS	135 steroidal saponins,47 homoiso flavonoids and 9 other metabolites were quickly identified or tentatively identified	^[67]^
Geographical	*Pseudostellariae* Radix (PR) in traditional fields (Jurong, Jiangsu, JSJR) and cultivated fields (Zherong, Fujian, FJZR)	HPLC, ^1^H-NMR	34 metabolites were identified, and 14 of them were significantly different in PR from different cultivated fields	^[68]^
Geographical and parts	Different parts of *G uralensis* from 5 different geographical origins in China	UHPLC-MS	16 potential biomarkers of taproots from different geographical origins were annotated	^[69]^
Geographical	Seedlings of S salsa were cultured with 0, 200 and 500 mM NaCl	UPLC-MS	A total of 639 metabolites were annotated. Among these, 253 metabolites were differential metabolites	^[70]^
Parts	The root and aerial parts of Bupleurum species	UHPLC-MS	A total of 56 peaks were identified in the root and/or aerial parts from different batches of Bupleurum species, by comparison of references standards or with those reported in the literature	^[71]^
Parts	Mahuang (MH, the stems of *Ephedra sinica*) and Mahuanggen (MHG, the roots of *Ephedra sinica*)	GC-MS	32 differential chemical markers were identified, and The contents of 2 pharmacologically important chemical markers of α-terpineol and TMP were significantly higher in most MH samples than that in MHG samples	^[72]^

CD = *Cistanche deserticola*, CT = *Cistanche tubulosa*, CMTP = Chinese mid-temperate propolis, CWTP = Chinese warm temperate propolis, FF = Farfarae Flos, MS = mass spectrometry, NMR = nuclear magnetic resonance, TCH = traditional Chinese herbs, UPLC = ultra performance liquid chromatography.

### 4.2. Safety evaluation

Traditional Chinese herbs (TCHs) have played a crucial role in treating diseases and global health maintenance for centuries. Given the chemical diversity and complexity of TCHs, where multiple bioactive constituents contribute synergistically to therapeutic effects, conventional toxicity evaluation methods often fail to elucidate specific molecular mechanisms. Consequently, safety concerns regarding TCHs persist, constituting a major bottleneck that impedes their modernization and internationalization. To address this, holistic omics approaches (including genomics, metabolomics, and proteomics) have been increasingly employed for toxicity assessment. Current applications of omics technologies in TCH safety research are systematically summarized in Table 2.^[73–91]^

**Table 2:** Omics technology studies on safety of TCHs and prescription.

TCHs	Toxicity	Samples	Technology	Result	References
*Dysosma versipellis*	Hepatotoxicity	Serum of rats	LC-MS	There were found to be 5 potentially toxic components. These compounds regulate PAH, SOD1, SOD2, and other related targets, which in turn causes oxidative stress, cell apoptosis, inflammatory response, and energy consumption, all of which lead to the development of liver injury. They also affect phenylalanine metabolism, glycerophospholipid metabolism, energy metabolism, and other related pathways	^[73]^
Cantharidin	Hepatotoxicity	Serum of mice	LC-MS	Four metabolites – oxidized glutathione, glutathione, 3-sulfinoalanine, and deoxycholic acid 3-glucuronide – were chosen and are crucial in liver injury out of the 54 substantially altered metabolites and 14 disrupted metabolic pathways that were found	^[74]^
Pinelliae Rhizoma	Cardiotoxicity	Serum of rats	LC-MS	10 significantly altered metabolites were identified	^[75]^
Arecae semen	Cardiotoxicity and neurotoxicity	Serum of rats	LC-MS	19 potential biomarkers were identified, they are related to the perturbations of linoleic acid metabolism, arachidonic acid metabolism, glycerophospholipid metabolism, and purine metabolism	^[76]^
Fructus Psoraleae	Hepatotoxicity	Serum of rats	SDS-PAGE and LC-MS	The problem of bile acid metabolism, oxidative stress, immune system, and disruption of energy metabolism are associated with bile secretion, glutathione metabolism, purine metabolism, glycerophospholipid metabolism, TCA cycle, and pyruvate metabolism	^[77]^
Aconitum	Cardiotoxicity and hepatotoxicity	Urine and serum of rats	LC-MS	Caowu primarily impacted the liver’s glucuronate interconversions, nucleotide sugar metabolism, fatty acid metabolism, and proline metabolism; it also had an impact on the heart’s taurine transformation, amino sugar metabolism, and tryptophan metabolism	^[78]^
Venenum Bufonis	Cardiotoxicity	Serum, myocardial and liver of rats	^1^H-NMR	The harmful effects of VB include mitochondrial malfunction, oxidative stress, disruption of energy metabolism, and disruption of membrane damage	^[79]^
*Euphorbia pekinensis*	Nephrotoxicity	Serum and kidney of rats	LC-MS	10 prototype components were found in the serum and 25 chemicals in the PE section were identified	^[80]^
Zhi-Zi-Hou-Po	Hepatotoxicity	Serum, the liver, lung, kidney, heart, brain, and spleen of rats	LC-MS	20 possible endogenous metabolites have been identified; geniposide buildup in liver tissue may be one of the causes of hepatotoxicity	^[81]^
Kan sui radix (KR),	Acute toxicity	Intestinal tract and serum of rats	LC-MS	The plasma contained identified biomarkers, such as phenol sulfate, indoxyl sulfate, and *p*-cresol sulfate, which were considerably disturbed in the rats. Only the contents of the colon and rectum contained the 3 necessary amino acids that generated these biomarkers	^[82]^
Polygonum Multiflorum	Hepatotoxicity	Serum and liver of rats	LC-MS and GC-MS	By using metabolomic analysis, a total of 12 distinct metabolites were identified. Seven metabolic pathways were examined in the topological study, which significantly disrupted the metabolism of bile acid, energy, and amino acids	^[83]^
*Tripterygium wilfordii* Hook	Cardiotoxicity	Serum and heart of rats	SDS-PAGE and LC-MS	It was shown that the TNF/caspase axis, which is regulated by oxidative stress created by palmitic acid, plays a part in the cardiotoxicity caused by CS	^[84]^
Dioscorea bulbifera rhizome	Hepatotoxicity	Serum and liver of rats	GC-MS and ^1^H-NMR	43 metabolic pathways contained the 61 metabolites that were tested as substantially changed metabolites. The hub metabolites of hepatotoxicity may be mostly associated with the metabolisms of amino acids, lipids, purines, pyrimidines, bile acid, gut microbiota, and energy, according to the correlation network analysis. Notably, in rats with DBR-induced liver injury, purine, pyrimidine, and gut microbiota metabolisms may be novel routes involved in metabolic abnormalities	^[85]^
Asarum	Hepatotoxicity	Liver of rats	GC-MS and DEG	Aside from the P53 signaling system, bile acid and amino acid metabolism disturbance may also play a role in asarum-induced hepatotoxicity	^[86]^
Xianling Gubao Capsule	Hepatotoxicity	Serum rats	LC-MS	XLGB susceptibility-related variables and biomarkers have been investigated	^[87]^
*Heterometrus spinifer*		Venomous glands	Next generation sequencing (NGS)	After a wealth of previously unidentified sequences were found and functional annotation was performed, a selection of toxic or venomous peptides was made. These included ion channel effectors like calcium and potassium channel toxins, as well as some peptides originating from scorpions like La1-like and scorpine-like peptides, antimicrobial peptides, protease inhibitors, and enzymes	^[88]^
Cinnabar	Neurotoxicity	Brain of rats	^1^H-NMR	Increased glutamate, glutamine, myoinositol, and choline levels, together with decreased levels of GABA, taurine, NAA, and NAAG in tissue extracts of the cerebellum and cerebrum, were the characteristics of the metabolite changes caused by cinnabar	^[89]^
Genkwa Flos	Hepatotoxicity	HL-7702 liver cells	UPLC-MS	9 biomarkers were found. Furthermore, oxidative stress and lipid peroxidation, the PLA2/LPC pathway, the disruption of the S1P metabolic profile, which is concentrated on the Sphk/S1P pathway, and fatty acid metabolism may all play a significant role in the development of liver injury brought on by GF	^[90]^
Aristolochic acid	Nephrotoxicity	Urine of rats	UPLC QTOF/HRMS	Changes in the Krebs cycle, gut microbiota metabolism, amino acid metabolism, purine metabolism, and bile acid production were seen in rats exposed to aristolochic acid nephrotoxicity	^[91]^

HRMS = high-resolution mass spectrometry, MS = mass spectrometry, NGS = next generation sequencing, NMR = nuclear magnetic resonance, TCH = traditional Chinese herbs, UPLC = ultra performance liquid chromatography.

#### 4.2.1. Hepatotoxicity

*Dysosma versipellis* exhibits multiple therapeutic functions including phlegm elimination, detoxification, blood stasis removal, and nodule dispersion. Despite its well-known potent cytotoxic and neurotoxic characteristics, the specific toxic ingredients and underlying mechanism remain equivocal. Liu et al^[73]^ employed ultra performance liquid chromatography (UPLC)-Q-TOF/high-resolution mass spectrometry coupled with network toxicology to investigate critical toxicity events, adverse outcomes, and hepatotoxic components in *D versipellis*. Their findings identified 5 potentially hazardous compounds: podophyllotoxin-4-*O*-d-glucoside, podorhizol, podophyllotoxin, podophyllotoxone, and 3,4-*O*,*O*-Didemethylpophyllotoxin. These constituents regulate targets (e.g., PAH, SOD1, SOD2), disrupting phenylalanine, glycerophospholipid, and energy metabolism pathways. Consequently, they induce oxidative stress, apoptosis, inflammation, and energy depletion, collectively contributing to liver injury. LC-MS technology has been applied not only to hepatotoxicity studies of *Fructus cantharidin*,^[74]^ Caowu,^[78]^ *Polygonum multiflorum* (PM),^[83]^ and *Genkwa flos*,^[90]^ but also to the Xianling Gubao Capsule.^[87]^ Zhao et al^[85]^ utilized GC-MS and ^1^H-NMR to characterize molecular alterations associated in *Dioscorea bulbifera rhizome*-induced subchronic hepatotoxicity, identifying 61 significantly altered metabolites across 43 pathways. Correlation network analysis revealed that gut microbiota metabolism, lipid metabolism, and purine/pyrimidine, bile acid, amino acid, and energy metabolisms constitute primary connections among hepatotoxicity hub metabolites. Duan^[77]^ further Fructus psoraleae hepatotoxicity through integrated proteomics-metabolomics technique, identifying fourteen critical metabolites and pathways related to immune response, oxidative stress, and bile acid/energy metabolism.

#### 4.2.2. Cardiotoxicity

*Pinelliae rhizoma* (PR) exhibits antitussive, antiemetic, expectorant, and antitumor activities.^[92,93]^ However, it contains teratogenic, carcinogenic, mutagenic, and reproductive toxins.^[94]^ Su et al^[95]^ investigated the mechanisms underlying raw PR-induced cardiotoxicity through LC-TOF-MS analysis of rat serum metabolites. Their study identified involvement of 2 molecular pathways (mTOR and TGF-β signaling) in PR-induced cardiotoxicity. LC-MS-based cardiotoxicity studies have been successfully for *Arecae semen*,^[76]^ Caowu^[78]^ and *celastrol*.^[84]^ In order to investigate the cardiotoxicity of venenum bufonis in rats, Dong et al^[79]^ employed ^1^H-NMR combined with behavioral observation, clinical chemistry, electrocardiography recordings, and histopathology. Results demonstrated that the heart damage caused by venenum bufonis is linked to oxidative stress, mitochondrial dysfunction, and disruptions in energy metabolism.

#### 4.2.3. Nephrotoxicity

*Euphorbia pekinensis* Rupr. (Euphorbiaceae; EPR) is traditionally used to treat edema, scrofula and various inflammation. Current pharmacological research has revealed that EPR also disperses phlegm, facilitates fluid purging, and reduces oppilation through purgative effects.^[96]^ However, clinical application of EPR is limited by its cytotoxic activity against human cells in vitro.^[97]^ Toxicology studies indicate that excessive doses and prolonged use induced hepatotoxicity and nephrotoxicity, although the detailed mechanism remian unclear. Liu et al^[80]^ investigated the serum metabolic parameters associated with EPR-induced nephrotoxicity using HPLC/Q-TOF-MS, aiming to correlate metabonomic profiling with histopathologic observations and identify biomarkers. Their results demonstrated disruptions in purine, amino acid, phospholipid, and sphingolipid metabolic pathways. Twenty-five compounds were identified in the processed EPR section, with an additional ten detected in rat serum post-administration. Omics technology has also been applied to elucidate nephrotoxic compounds and pathways in other botanicals, such as aristolochic acid and *Morning glory* Seed.^[98]^

#### 4.2.4. Other toxicity

Clinical applications of Kansui radix are hindered by its acute toxicity. Yang et al^[82]^ studied rat plasma and intestinal contents are studied using the LC-MS technology to investigate the toxicity mechanism. P-cresol sulfate, indoxyl sulfate, and phenol sulfate are colon-derived uremic chemicals that were previously considered harmful to the kidney, heart, vascular, and neurological systems. Cinnabar, which contains more than 95% HgS, is commonly used for sedative therapy. However, its clinical application is limited by neurotoxicity. To address this issue, Wei et al^[89]^ employed ^1^H-NMR spectroscopy to study cinnabar’s neurotoxic effects, analyzing extracts from cerebrum and cerebellum tissues samples that yield homogenous, macromole-free solutions with low viscosity and narrow spectral peaks.

### 4.3. Processing

The processing of TCM, called Pao Zhi, which is built on the dialectical treatments and the characteristics of TCM, has not only altered the TCM properties and increased clinical efficacy, but also reduced side effects.^[99,100]^ It has been well demonstrated that the processing method can change the contents and varieties of chemical composition, and alter the inclination and direction of TCM’s therapeutic effects. For instance, a study suggested utilizing ultra-high-performance liquid chromatography-MS to simultaneously determine and assess the quality of sixteen components in Ligustri Lucidi Fructus (LLF) that cover various locations and processed products.^[101]^ Furthermore, the effects of wine-processing on ascending and descending were studied. The rat upper-energizer tissues (lung and heart) showed a notable increase in the parameters of some flavonoids in wine-processed Radix scutellariae, while the rat middle- and lower-energizer tissues (spleen, liver, and kidney) showed a significant decrease in some flavonoids when compared to the crude group.^[102]^ This research, however, is lacking since the chemical profile of TCM was not fully represented; instead, only a small number of common chemical components were selected. Recently, omics technology has been widely used to thoroughly investigate the variations in chemicals in TCM, including processing.

#### 4.3.1. Change of components

The first step in establishing a scientific basis for TCM processing is to investigate how processing method alter the integrated chemical compositions. Liang et al^[103]^ employed targeted and untargeted metabolomics using UPLC-QTOF-MS/MS and UPLC-QqQ-MS/MS in an integrated approach to study the chemistry of *Polygoni multiflflori* radix after processing. The chemical composition differed significantly, and the researchers discovered fewer potentially disruptive compounds. These findings imply that the omics technology can provide a thorough characterization of TCM’s chemical profile after processing, which helps clarify the scientific foundation of processing methods. Additionally, using the same comprehensive technique, differences in the glycome and metabolome of *Rehmanniae* Radix (RR) across 9 cycles of steaming and drying were identified.^[104,105]^ Furthermore, omics technology combined with other strategies can reveal the global chemical changes involved in TCM between raw and processed forms. For example, a metabolomics strategy incorporating multicomponent characterization and MS imaging was used to reveal the processing-induced holistic chemical changes in LLF after wine steaming.^[106]^ The most fundammental step in TCM processing is drying, which significantly impacts components and quality. Chemical changes in fresh Notopterygium franchetii during various drying methods (sun, shade, microwave, oven, freezer, and infrared) were examined using a UHPLC-DAD-QTOF-MS/MS. Concentrations of 6 primary bioactive elements were greatly reduced with all techniques. Moreover, shade drying resulted in the most chemical alterations, while hot air drying produced the fewest changes at the lowest cost.^[107]^ To investigate the characteristics of raw and processed RR and identify active ingredients responsible for their differences, metabolicomics and network pharmacology were integrated.^[108,109]^ Untargeted metabolomics combined with MS and multicomponent characterization revealed key markers and comprehensive chemical changes associated with wine steaming of LLF.^[106]^ Targeted glycomics and untargeted metabolomics demonstrated that the glycome and metabolome of RR varied during 9 steaming and drying cycles. By examining the processing chemistry in both raw and processed RR, researchers clarified chemical transformation mechanisms of carbohydrates and secondary metabolites induced by processing.^[105]^ Dai et al^[110]^ and Li et al^[109]^ investigated quality marker identification using ^1^H-NMR- and electrospray ionization-QTOFMS-based metabolomics on standard decoction of differently processed Ephedrae Herba. This investigation demonstrated that examining chemical indicators correlated with TCM’s therapeutic efficacy through conventional decoction is a valid research objective.

#### 4.3.2. Increase of the potency

Most TCHs, when subjected to reasonable processing methods, can enhance pharmacological activity before clinical use. Using UHPLC-QTOF/MS-based plasma metabolomics, Zhang et al^[111]^ studied the effects of both raw and bran-fried Atractylodis Rhizoma on the same metabolites in spleen deficiency, providing a comprehensive explanation of the processing mechanism of Atractylodis Rhizoma.

The inflammatory exudate and plasma samples from model rats in the stir-fried *Angelica sinensis* (Oliv.) Diels group, alcohol-parched Angelica sinensis group, soil-parched Angelica sinensis group, and sesame oil-parched Angelica sinensis group were analyzed using GC-MS based metabolomics to identify metabolites. The results demonstrated that the anti-inflammatory effects of these VOAS groups could be distinctly differentiated and categorized *via* metabolite-based profiling. Alcohol-parched Angelica sinensis group, Diels group, sesame oil-parched Angelica sinensis group, and soil-parched Angelica sinensis group differed in their abilities to suppress inflammatory mediators release, modulate potential biomarke concentration, and regulate disrupted pathways.^[112]^ Mechanisms technologies based on LC-Q/TOF-MS also elucidated the mechanisms of Angelica sinensis and its processed products in promoting blood circulation and alleviating blood stasis.^[113]^ Additionally, a combined metabolomic and metallomic approach using GC-MS and ICP-MS effectively assessed the anti-osteoporosis effects of raw and salt-processed *Achyranthes bidentata blume* on ovariectomized rats.^[114]^ Untargeted metabolomics integrated with targeted analysis compared the antidiabetic effects and chemical profiles of raw and fermented Ge-Gen-Qin-Lian decoction.^[115]^ Sulfur-fumigation impacts on the pharmacokinetics, metabolites, and analgesic efficacy of Radix Paeoniae Alba metabolomics were examined through metabolomics.^[116]^ GC/MS-based metabolomics and multivariate statistical analysis investigated the pharmacological effects of fresh, dried, and stir-fried ginger decoctions in rats with blood stasis syndrome.^[117]^ Collectively, omics approaches facilitate understanding how processing enhances TCM effectiveness.

#### 4.3.3. Reduction of toxicity

There have been reports of extremely harmful consequences from some TCM, and toxicity may vary depending on the processing method used. Utilizing multivariate statistical analysis and UHPLC/Q-Orbitrap-MS, Han et al^[118]^ examined the differences in PM metabolite profiles caused by various processing technologies. It was revealed that the optimal processing method for PM was steaming in black soybean decoction for 24 hours. Torchrysone-*O*-hexose and emodin-8-*O*-glucoside were employed as potential toxic indicators to evaluate different processing methods. Aconiti kusnezoffii radix was tested for nephrotoxicity and neurotoxicity using metabolomics with serum biochemistry and histopathology. This approach was shown to be a potent means of examining the mechanisms underlying herbal toxicity and its reduction through processing. Furthermore, it may offer safe alternatives to harmful herbal remedies and provide additional evidence for how Aconiti kusnezoffii radix cocta cloud alleviate symptoms in rheumatoid arthritis patients.

### 4.4. Authentication

Due to similar physical traits, many plants have long been difficult to authenticate. When the pharmaceutical industry uses incorrect plant materials, this can cause complex issues for clinical practice and TCM product manufacturing. Zhang et al developed an integrated strategy platform based on chemometrics to characterize and categorize 4 Pulsatilla Adans herbs, combining UHPLC/Q-Orbitrap-MS techniques with chemometrics analysis.^[119]^ Known in Chinese as Dong Chong Xia Cao (DCXC), Ophiocordyceps sinensis (syn. *Cordyceps sinensis*) is a well-known TCM with various health-promoting benefits. Nonetheless, imitations and fakes of DCXC are commonly found in marketplaces. To authenticate DCXC, several researchers^[120]^ employed metabolomics technologies, including ^1^H-NMR and quadrupole time-of-flight MS, integrated with ultrafast liquid chromatography. The findings indicated that metabolomics holds considerable promise for the rapid identification of DCXC adulteration.

## 5. Conclusions and prospects

The sustainable development of TCM fundamentally relies on product quality assurance. To ensure TCM’s safety, effeccy, stability, and quality controllability, collaborative efforts between academia and industry are essential. Consequently, establishing a quality control system that both aligns with TCM’s theoretical principles and gains international pharmaceutical recognition becomes imperative. While conventional postproduction testing methods have become obsolete due to their reactive nature, the implementation of Q-markers – characterized by traceability and transitivity-represents a paradigm shift toward proactive quality management. Three major challenges currently hinder TCM advancement, insufficient understanding of processing mechanism-of-action pathways, persistent raw material adulteration issues, and unidentified bioactive constituents. Particularly for animal-derived TCMs, active components often comprise small molecules while the predominant chemical profiles consist of complex macromolecules (e.g., proteins and peptides) that pose analytical difficulties. Furthermore, conservation policies restricting wild medicinal resource utilization create supply chain bottlenecks, necessitating the development of synthetic alternatives to natural medicinal materials. Addressing these multidimensional challenges requires breakthroughs in basic research through advanced technological integration, including but not limited to omics technologies, AI-assisted analysis, and advanced separation techniques.

The “omics age”-marked by the ability to collect and integrate data across many molecular modalities-has emerged through technological developments. The advent of systems biology-driven omics analysis has significantly advanced TCM research. This shift implies that TCM studies have transitioned from conventional biochemical and molecular analysis methods to data-driven omics approaches.

To effectively regulate the entire production process, Q-markers should optimally represent the efficacy, safety, and consistent quality of TCM. It is critical to note that not all active ingredients quality as suitable Q-markers, and Q-markers do not simply equal the sum of quality indicators for individual herbs in a formulation. The identification of novel biomarkers and development of advanced testing methodologies remain pivotal in TCM research. Enhanced DNA methylation studies are required to refine TCM interpretation and quality assessment. While proteomics has gained significant attention in TCM research-particularly for animal-derived products due to its reproducible and verifiable results-its application is constrained by high costs associated with testing and reagents.

## Author contributions

**Conceptualization:** Jinglin Shen.

**Resources:** Yiming Che.

**Software:** Jinjin Wu.

**Writing – original draft:** Chenqiong Xie.

**Writing** – **review & editing:** Yanxia Zhou, Jinglin Shen.

## References

[1] Wu SX Su WQ Fan QH Shang HC Xiao W Wang YY. Traditional Chinese medicine for the common cold: evidence and potential mechanisms. Am J Chin Med. 2023;51:487–515. 36803206 10.1142/S0192415X23500258

[2] Jin Q Liu TT Ma F. Therapeutic application of traditional Chinese medicine in kidney disease: sirtuins as potential targets. Biomed Pharmacother. 2023;167:115499. 37742600 10.1016/j.biopha.2023.115499

[3] Lu XY Jin YY Wang YZ Chen YL Fan XH. Multimodal integrated strategy for the discovery and identification of quality markers in traditional Chinese medicine. J Pharm Anal. 2022;12:701–10. 36320607 10.1016/j.jpha.2022.05.001 PMC9615540

[4] Babu M Snyder M. Multi-omics profiling for health. Mol Cell Proteomics. 2023;22:100561. 37119971 10.1016/j.mcpro.2023.100561 PMC10220275

[5] Muthubharathi BC Gowripriya T Balamurugan K. Metabolomics: small molecules that matter more. Mol Omics. 2021;17:210–29. 33598670 10.1039/d0mo00176g

[6] Bock DG Cai Z Elphinstone C. Genomics of plant speciation. Plant Commun. 2023;4:100599. 37050879 10.1016/j.xplc.2023.100599 PMC10504567

[7] Aslam B Basit M Nisar MA Khurshid M Rasool MH. Proteomics: technologies and their applications. J Chromatogr Sci. 2017;55:182–96. 28087761 10.1093/chromsci/bmw167

[8] Gilhooley MJ Owen N Moosajee M Yu Wai Man P. From transcriptomics to treatment in inherited optic neuropathies. Genes (Basel). 2021;12:147. 33499292 10.3390/genes12020147 PMC7912133

[9] Guo R Luo X Liu J Liu L Wang X Lu H. Omics strategies decipher therapeutic discoveries of traditional Chinese medicine against different diseases at multiple layers molecular-level. Pharmacol Res. 2020;152:104627. 31904505 10.1016/j.phrs.2020.104627

[10] Emboden W. Narcotic Plants. Macmillan Publishing Co; 1979.

[11] Chen KJ Li CS. Xinbian Kang Shuailao Zhongyaoxue. People’s Health Publishing House; 1998.

[12] Yan ZZ Zhong L Zhu WD Chung SK Hou PP. Chinese herbal medicine for the treatment of cardiovascular diseases -targeting cardiac ion channels. Pharmacol Res. 2023;192:106765–1186. 37075871 10.1016/j.phrs.2023.106765

[13] Wang X Li J Chen R Li T Chen MW. Active ingredients from Chinese medicine for combination cancer therapy. Int J Biol Sci. 2023;19:3499–525. 37497002 10.7150/ijbs.77720 PMC10367560

[14] Hao DL Li JM Xie R. The role of traditional herbal medicine for ischemic stroke: from bench to clinic-a critical review. Phytomedicine. 2023;109:154609. 36610141 10.1016/j.phymed.2022.154609

[15] Li W Yu L Li W. Prevention and treatment of inflammatory arthritis with traditional Chinese medicine: underlying mechanisms based on cell and molecular targets. Ageing Res Rev. 2023;89:101981. 37302756 10.1016/j.arr.2023.101981

[16] Lyu M Fan G Xiao G. Traditional Chinese medicine in COVID-19. Acta Pharm Sin B. 2021;11:3337–63. 34567957 10.1016/j.apsb.2021.09.008 PMC8450055

[17] Zhao ZY Li YD Zhou LY. Prevention and treatment of COVID-19 using traditional Chinese medicine: a review. Phytomedicine. 2021;85:153308. 32843234 10.1016/j.phymed.2020.153308 PMC7439087

[18] Zhang H Zhang Y Zhang T Liu C. Research progress on quality markers of traditional Chinese medicine. J Pharm Biomed Anal. 2022;211:114588. 35091155 10.1016/j.jpba.2022.114588

[19] Lu X Jin Y Wang Y Chen Y Fan X. Multimodal integrated strategy for the discovery and identification of quality markers in traditional Chinese medicine. J Pharm Anal. 2022;12:701–10. 36320607 10.1016/j.jpha.2022.05.001 PMC9615540

[20] Madden EB Hindorff LA Bonham VL. Advancing genomics to improve health equity. Nat Genet. 2024;56:752–7. 38684898 10.1038/s41588-024-01711-z PMC11096049

[21] Hamilton JP Robin Buell C. Advances in plant genome sequencing. Plant J. 2012;70:177–90. 22449051 10.1111/j.1365-313X.2012.04894.x

[22] Chen F Dong W Zhang J. The sequenced angiosperm genomes and genome databases. Front Plant Sci. 2018;9:418. 29706973 10.3389/fpls.2018.00418 PMC5909171

[23] Jiao WB Schneeberger K. The impact of third generation genomic technologies on plant genome assembly. Curr Opin Plant Biol. 2017;36:64–70. 28231512 10.1016/j.pbi.2017.02.002

[24] Chen SL Sun YZ Xu J. Strategies of the study on Herb genome program (草本植物基因组计划研究策略). Yao Xue Xue Bao. 2010;45:807–12. 20931775

[25] Chen SL Wu WG Wang CX. Molecular genetics research of medicinal plants (药用植物分子遗传学研究). Zhongguo Zhong Yao Za Zhi. 2019;44:2421–32. 31359707 10.19540/j.cnki.cjcmm.20190514.102

[26] Yasen A Aini A Wang H. Progress and applications of single-cell sequencing techniques. Infect Genet Evol. 2020;80:104198. 31958516 10.1016/j.meegid.2020.104198

[27] Shojaee A Saavedra M Huang SC. Potentials of single-cell genomics in deciphering cellular phenotypes. Curr Opin Plant Biol. 2021;63:102059. 34116424 10.1016/j.pbi.2021.102059 PMC8545747

[28] Tenorio Berrio R Verstaen K Vandamme N. Single-cell transcriptomics sheds light on the identity and metabolism of developing leaf cells. Plant Physiol. 2022;188:898–918. 34687312 10.1093/plphys/kiab489 PMC8825278

[29] Chen C Ge Y Lu L. Opportunities and challenges in the application of single-cell and spatial transcriptomics in plants. Front Plant Sci. 2023;14:1185377. 37636094 10.3389/fpls.2023.1185377 PMC10453814

[30] Tu Y Tan L Tao H Li Y Liu H. CETSA and thermal proteome profiling strategies for target identification and drug discovery of natural products. Phytomedicine. 2023;116:154862. 37216761 10.1016/j.phymed.2023.154862

[31] Valdés A García-Cañas V Artemenko KA Simó C Bergquist J Cifuentes A. Nano-liquid chromatography-orbitrap ms-based quantitative proteomics reveals differences between the mechanisms of action of carnosic acid and carnosol in colon cancer cells. Mol Cell Proteomics. 2017;16:8–22. 27834734 10.1074/mcp.M116.061481 PMC5217784

[32] Nicholson JK Lindon JC Holmes E. Metabonomics: understanding the metabolic responses of living systems to pathophysiological stimuli via multivariate statistical analysis of biological NMR spectroscopic data. Xenobiotica. 1999;29:1181–9. 10598751 10.1080/004982599238047

[33] DeBerardinis RJ Keshari KR. Metabolic analysis as a driver for discovery, diagnosis, and therapy. Cell. 2022;185:2678–89. 35839759 10.1016/j.cell.2022.06.029 PMC9469798

[34] Armand EJ Li J Xie F Luo C Mukamel EA. Single-cell sequencing of brain cell transcriptomes and epigenomes. Neuron. 2021;109:11–26. 33412093 10.1016/j.neuron.2020.12.010 PMC7808568

[35] Pandian K Matsui M Hankemeier T Ali A Okubo-Kurihara E. Advances in single-cell metabolomics to unravel cellular heterogeneity in plant biology. Plant Physiol. 2023;193:949–65. 37338502 10.1093/plphys/kiad357 PMC10517197

[36] Liu C Zhang X Nguyen TT. Partial least squares regression and principal component analysis: similarity and differences between two popular variable reduction approaches. Gen Psychiatr. 2022;35:e100662. 35146334 10.1136/gpsych-2021-100662 PMC8796256

[37] Domingo-Almenara X Siuzdak G. Metabolomics data processing using XCMS. Methods Mol Biol. 2020;2104:11–24. 31953810 10.1007/978-1-0716-0239-3_2

[38] Schmid R Heuckeroth S Korf A. Integrative analysis of multimodal mass spectrometry data in MZmine 3. Nat Biotechnol. 2023;41:447–9. 36859716 10.1038/s41587-023-01690-2 PMC10496610

[39] LaPierre N Alser M Eskin E Koslicki D Mangul S. Metalign: efficient alignment-based metagenomic profiling via containment min hash. Genome Biol. 2020;21:242. 32912225 10.1186/s13059-020-02159-0 PMC7488264

[40] Baran R Kochi H Saito N. MathDAMP: a package for differential analysis of metabolite profiles. BMC Bioinf. 2006;7:530. 10.1186/1471-2105-7-530 PMC1764210 17166258

[41] Wang L Xing X Zeng X. Spatially resolved isotope tracing reveals tissue metabolic activity. Nat Methods. 2022;19:223–30. 35132243 10.1038/s41592-021-01378-y PMC10926149

[42] Zhang W Jiang H Yang J. A high-throughput metabolomics approach for the comprehensive differentiation of four Pulsatilla Adans herbs combined with a nontargeted bidirectional screen for rapid identification of triterpenoid saponins. Anal Bioanal Chem. 2019;411:2071–88. 30734858 10.1007/s00216-019-01631-6

[43] Li SS Wu Q Yin DD Feng CY Liu ZA Wang LS. Phytochemical variation among the traditional Chinese medicine Mu Dan Pi from Paeonia suffruticosa (tree peony). Phytochem. 2018;146:16–24. 10.1016/j.phytochem.2017.11.008 29207319

[44] Tao HX Xiong W Zhao GD. Discrimination of three Siegesbeckiae Herba species using UPLC-QTOF/MS-based metabolomics approach. Food Chem Toxicol. 2018;119:400–6. 29305931 10.1016/j.fct.2017.12.068

[45] Song YL Song QQ Li J. An integrated strategy to quantitatively differentiate chemome between Cistanche deserticola and C. tubulosa using high performance liquid chromatography-hybrid triple quadrupole-linear ion trap mass spectrometry. J Chromatogr A. 2016;1429:238–47. 26742897 10.1016/j.chroma.2015.12.045

[46] Tanaka K Arita M Sakurai H Ono N Tezuka Y. Analysis of chemical properties of edible and medicinal ginger by metabolomics approach. Biomed Res Int. 2015;2015:671058. 26495311 10.1155/2015/671058 PMC4606115

[47] Guo LX Li R Liu K. Structural characterization and discrimination of Chinese medicinal materials with multiple botanical origins based on metabolite profiling and chemometrics analysis: clematidis radix et rhizoma as a case study. J Chromatogr A. 2015;1425:129–40. 26610614 10.1016/j.chroma.2015.11.013

[48] Chen L Huang X Wang H. Integrated metabolomics and network pharmacology strategy for ascertaining the quality marker of flavonoids for Sophora flavescens. J Pharm Biomed Anal. 2020;186:113297. 32325403 10.1016/j.jpba.2020.113297

[49] Xing D Shen G Li Q Xiao Y Yang Q Xia Q. Quality formation mechanism of stiff silkworm, bombyx batryticatus using UPLC-Q-TOF-MS-based metabolomics. Molecules. 2019;24:3780. 31640173 10.3390/molecules24203780 PMC6832393

[50] Lv MY Sun JB Wang M Fan HY Zhang ZJ Xu FG. Comparative analysis of volatile oils in the stems and roots of Ephedra sinica via GC-MS-based plant metabolomics. Chin J Nat Med. 2016;14:133–40. 26968679 10.1016/S1875-5364(16)60006-7

[51] Li J Zhang ZZ Lei ZH Qin XM Li ZY. NMR based metabolomic comparison of the antitussive and expectorant effect of Farfarae Flos collected at different stages. J Pharm Biomed Anal. 2018;150:377–85. 29287265 10.1016/j.jpba.2017.12.028

[52] Wang T Liu Q Wang M Zhang L. Metabolomics reveals discrimination of chinese propolis from different climatic regions. Foods. 2020;9:491. 32295098 10.3390/foods9040491 PMC7230208

[53] Hua Y Hou Y Wang S. Comparison of chemical compositions in pseudostellariae radix from different cultivated fields and germplasms by NMR-based metabolomics. Molecules. 2016;21:1538. 27854294 10.3390/molecules21111538 PMC6273876

[54] Li Z Du Y Yuan Y Zhang X Wang Z Tian X. Integrated quality evaluation strategy for multi-species resourced herb medicine of Qinjiao by metabolomics analysis and genetic comparation. Chin Med. 2020;15:16. 32071612 10.1186/s13020-020-0292-3 PMC7014644

[55] Tao HX Xiong W Zhao GD. Discrimination of three Siegesbeckiae Herba species using UPLC-QTOF/MS-based metabolomics approach. Food Chem Toxicol. 2018;119:400–6. 29305931 10.1016/j.fct.2017.12.068

[56] Song Y Song Q Li J. An integrated strategy to quantitatively differentiate chemome between Cistanche deserticola and C. tubulosa using high performance liquid chromatography-hybrid triple quadrupole-linear ion trap mass spectrometry. J Chromatogr A. 2016;1429:238–47. 26742897 10.1016/j.chroma.2015.12.045

[57] Tanaka K Arita M Sakurai H Ono N Tezuka Y. Analysis of chemical properties of edible and medicinal ginger by metabolomics approach. Biomed Res Int. 2015;2015:671058. 26495311 10.1155/2015/671058 PMC4606115

[58] Li SS Wu Q Yin DD Feng CY Liu ZA Wang LS. Phytochemical variation among the traditional Chinese medicine Mu Dan Pi from Paeonia suffruticosa (tree peony). Phytochem. 2018;146:16–24. 10.1016/j.phytochem.2017.11.008 29207319

[59] Lv MY Sun JB Wang M Fan HY Zhang ZJ Xu FG. Comparative analysis of volatile oils in the stems and roots of Ephedra sinica via GC-MS-based plant metabolomics. Chin J Nat Med. 2016;14:133–40. 26968679 10.1016/S1875-5364(16)60006-7

[60] Booker A Zhai L Gkouva C Li S Heinrich M. From traditional resource to global commodities:-a comparison of rhodiola species using NMR spectroscopy-metabolomics and HPTLC. Front Pharmacol. 2016;7:254. 27621703 10.3389/fphar.2016.00254 PMC5002433

[61] Zhao SY Liu ZL Shu YS. Chemotaxonomic classification applied to the identification of two closely-related citrus TCMs using UPLC-Q-TOF-MS-based metabolomics. Molecules. 2017;22:1721. 29027971 10.3390/molecules22101721 PMC6151587

[62] Guo LX Li R Liu K. Structural characterization and discrimination of Chinese medicinal materials with multiple botanical origins based on metabolite profiling and chemometrics analysis: Clematidis Radix et Rhizoma as a case study. J Chromatogr A. 2015;1425:129–40. 26610614 10.1016/j.chroma.2015.11.013

[63] Liu F Bai X Yang FQ. Discriminating from species of Curcumae Radix (Yujin) by a UHPLC/Q-TOFMS-based metabolomics approach. Chin Med. 2016;11:21. 27134643 10.1186/s13020-016-0095-8 PMC4850745

[64] Pan Z Xiong F Chen YL. Traceability of geographical origin in gentiana straminea by UPLC-Q exactive mass and multivariate analyses. Molecules. 2019;24:4478. 31817679 10.3390/molecules24244478 PMC6943584

[65] Zhang G Li Y Wei W. Metabolomics combined with multivariate statistical analysis for screening of chemical markers between gentiana scabra and gentiana rigescens. Molecules. 2020;25:1228. 32182812 10.3390/molecules25051228 PMC7179410

[66] Wu J Yang W Pan H Yao S Wu W Guo D. Geographic impact evaluation of the quality of Alismatis Rhizoma by untargeted metabolomics and quantitative assay. J Sep Sci. 2018;41:839–46. 29178373 10.1002/jssc.201700902

[67] Lyu CG Kang CZ Kang LP. Structural characterization and discrimination of Ophiopogon japonicas (Liliaceae) from different geographical origins based on metabolite profiling analysis. J Pharm Biomed Anal. 2020;185:113212. 32143114 10.1016/j.jpba.2020.113212

[68] Hua YJ Hou Y Wang SN. Comparison of chemical compositions in pseudostellariae radix from different cultivated fields and germplasms by NMR-based metabolomics. Molecules. 2016;21:1538. 27854294 10.3390/molecules21111538 PMC6273876

[69] Bai H Bao F Fan X. Metabolomics study of different parts of licorice from different geographical origins and their anti-inflammatory activities. J Sep Sci. 2020;43:1593–602. 32032980 10.1002/jssc.201901013

[70] Li Q Song J. Analysis of widely targeted metabolites of the euhalophyte Suaeda salsa under saline conditions provides new insights into salt tolerance and nutritional value in halophytic species. BMC Plant Biol. 2019;19:388. 31492100 10.1186/s12870-019-2006-5 PMC6729093

[71] Zhu L Liang ZT Yi T. Comparison of chemical profiles between the root and aerial parts from three Bupleurum species based on a UHPLC-QTOF-MS metabolomics approach. BMC Complement Altern Med. 2017;17:305. 28606186 10.1186/s12906-017-1816-y PMC5468949

[72] Xin GZ Hu B Shi ZQ. A direct ionization mass spectrometry-based approach for differentiation of medicinal Ephedra species. J Pharm Biomed Anal. 2016;117:492–8. 26473988 10.1016/j.jpba.2015.09.032

[73] Liu C Zhang C He T. Study on potential toxic material base and mechanisms of hepatotoxicity induced by Dysosma versipellis based on toxicological evidence chain (TEC) concept. Ecotoxicol Environ Saf. 2020;190:110073. 31851898 10.1016/j.ecoenv.2019.110073

[74] Zhu SS Long R Song T. UPLC-Q-TOF/MS based metabolomics approach to study the hepatotoxicity of cantharidin on mice. Chem Res Toxicol. 2019;32:2204–13. 31617706 10.1021/acs.chemrestox.9b00233

[75] Wang J Cui J Liu Z Yang Y Li Z Liu H. Untargeted metabolomics based on ultra-high-performance liquid chromatography coupled with quadrupole orbitrap high-resolution mass spectrometry for differential metabolite analysis of pinelliae rhizoma and its adulterants. Molecules. 2024;29:2155. 38731650 10.3390/molecules29092155 PMC11085193

[76] Lin Q Wang C Jia Z. UPLC-HDMS-based on serum metabolomics reveals the toxicity of arecae semen. J Ethnopharmacol. 2020;247:112223. 31553926 10.1016/j.jep.2019.112223

[77] Duan J Dong W Xie L Fan S Xu Y Li Y. Integrative proteomics-metabolomics strategy reveals the mechanism of hepatotoxicity induced by Fructus Psoraleae. J Proteomics. 2020;221:103767. 32240813 10.1016/j.jprot.2020.103767

[78] Yan Y Zhang A Dong H. Toxicity and detoxification effects of herbal caowu via ultra performance liquid chromatography/mass spectrometry metabolomics analyzed using pattern recognition method. Pharmacogn Mag. 2017;13:683–92. 29200734 10.4103/pm.pm_475_16 PMC5701412

[79] Dong G Wei D Wang J. Study of the cardiotoxicity of Venenum Bufonis in rats using an 1H NMR-based metabolomics approach. PLoS One. 2015;10:e0119515. 25781638 10.1371/journal.pone.0119515 PMC4363591

[80] Liu Z Zeng Y Hou P. Metabolomic evaluation of Euphorbia pekinensis induced nephrotoxicity in rats. Pharm Biol. 2018;56:145–53. 29421944 10.1080/13880209.2018.1435697 PMC6130632

[81] Wang Y Feng F. Evaluation of the hepatotoxicity of the Zhi-Zi-Hou-Po decoction by combining UPLC-Q-Exactive-MS-based metabolomics and HPLC-MS/MS-based geniposide tissue distribution. Molecules. 2019;24:511. 30708983 10.3390/molecules24030511 PMC6384998

[82] Yang Z Hou JJ Qi P. Colon-derived uremic biomarkers induced by the acute toxicity of Kansui radix: a metabolomics study of rat plasma and intestinal contents by UPLC-QTOF-MS(E). J Chromatogr B Analyt Technol Biomed Life Sci. 2016;1026:193–203. 10.1016/j.jchromb.2015.09.022 26433353

[83] Gong X Liu M Gong L Li Y Peng C. Study on hepatotoxicity of different dosages of Polygoni multiflori radix praeparata in rats by metabolomics based on UPLC-Q-TOF-MS. J Pharm Biomed Anal. 2019;175:112760. 31382117 10.1016/j.jpba.2019.07.008

[84] Liu C Zhang C Wang W. Integrated metabolomics and network toxicology to reveal molecular mechanism of celastrol induced cardiotoxicity. Toxicol Appl Pharmacol. 2019;383:114785. 31629732 10.1016/j.taap.2019.114785

[85] Zhao DS Wu ZT Li ZQ. Liver-specific metabolomics characterizes the hepatotoxicity of Dioscorea bulbifera rhizome in rats by integration of GC-MS and ^1^H-NMR. J Ethnopharmacol. 2018;226:111–9. 30114519 10.1016/j.jep.2018.08.014

[86] Cao S Han L Li Y. Integrative transcriptomics and metabolomics analyses provide hepatotoxicity mechanisms of asarum. Exp Ther Med. 2020;20:1359–70. 32742371 10.3892/etm.2020.8811 PMC7388312

[87] Li CY Niu M Liu YL. Screening for susceptibility-related factors and biomarkers of xianling gubao capsule-induced liver injury. Front Pharmacol. 2020;11:810. 32547402 10.3389/fphar.2020.00810 PMC7274038

[88] Deng Y Gu J Yan Z. De novo transcriptomic analysis of the venomous glands from the scorpion Heterometrus spinifer revealed unique and extremely high diversity of the venom peptides. Toxicon. 2018;143:1–19. 29305080 10.1016/j.toxicon.2017.12.051

[89] Wei L Xue R Zhang P Wu Y Li X Pei F. (1)H NMR-based metabolomics and neurotoxicity study of cerebrum and cerebellum in rats treated with cinnabar, a traditional Chinese medicine. OMICS. 2015;19:490–8. 26110755 10.1089/omi.2015.0042

[90] Wang Z Zhang Y Liu Q. Investigation of the mechanisms of Genkwa Flos hepatotoxicity by a cell metabolomics strategy combined with serum pharmacology in HL-7702 liver cells. Xenobiotica. 2019;49:216–26. 29325475 10.1080/00498254.2018.1427905

[91] Zhao YY Tang DD Chen H. Urinary metabolomics and biomarkers of aristolochic acid nephrotoxicity by UPLC-QTOF/HDMS. Bioanalysis. 2015;7:685–700. 25871586 10.4155/bio.14.309

[92] Luo W Ding R Guo X. Clinical data mining reveals Gancao-Banxia as a potential herbal pair against moderate COVID-19 by dual binding to IL-6/STAT3. Comput Biol Med. 2022;145:105457. 35366469 10.1016/j.compbiomed.2022.105457 PMC8957363

[93] Du Y Yan B Wang R. First report of pectobacterium aroidearum causing soft rot of pinellia ternata in China. Plant Dis. 2023;108:779.

[94] Peng W Li N Jiang E. A review of traditional and current processing methods used to decrease the toxicity of the rhizome of Pinellia ternata in traditional Chinese medicine. J Ethnopharmacol. 2022;299:115696. 36087845 10.1016/j.jep.2022.115696

[95] Su T Tan Y Tsui MS. Metabolomics reveals the mechanisms for the cardiotoxicity of Pinelliae Rhizoma and the toxicity-reducing effect of processing. Sci Rep. 2016;6:34692. 27698376 10.1038/srep34692 PMC5048190

[96] Wang K Xu X Shan Q. Integrated gut microbiota and serum metabolomics reveal the protective effect of oleanolic acid on liver and kidney-injured rats induced by Euphorbia pekinensis. Phytother Res. 2022;38:4877–92. 36426741 10.1002/ptr.7673

[97] Qin WN Zhang KC Geng T. The toxicity mechanism of toxic compounds from Euphorbiae pekinensis Radix on zebrafish embryos. Biomed Pharmacother. 2021;138:111521. 34311525 10.1016/j.biopha.2021.111521

[98] Ma C Bi K Su D. Serum and kidney metabolic changes of rat nephrotoxicity induced by Morning Glory Seed. Food Chem Toxicol. 2010;48:2988–93. 20678537 10.1016/j.fct.2010.07.038

[99] Pan L Wang Y Yue L. Review on processing methods of toxic Chinese materia medica and the related mechanisms of action. Am J Chin Med. 2023;51:1385–412. 37545180 10.1142/S0192415X23500635

[100] Wang Y Li P Zhang X. Mitochondrial-respiration-improving effects of three different gardeniae fructus preparations and their components. ACS Omega. 2021;6:34229–41. 34963909 10.1021/acsomega.1c03265 PMC8697009

[101] Li X Jia J Li T. Simultaneous determination and quality evaluation of 16 compounds in Ligustri Lucidi Fructus covering different regions and processed products using ultra-high-performance liquid chromatography-mass spectrometry. Biomed Chromatogr. 2023;37:e5564. 36509695 10.1002/bmc.5564

[102] Huang P Tan S Zhang YX. The effects of wine-processing on ascending and descending: The distribution of flavonoids in rat tissues after oral administration of crude and wine-processed Radix scutellariae. J Ethnopharmacol. 2014;155:649–64. 24930356 10.1016/j.jep.2014.05.063

[103] Liang L Xu J Zhou WW Brand E Chen HB Zhao ZZ. Integrating targeted and untargeted metabolomics to investigate the processing chemistry of polygoni multiflori radix. Front Pharmacol. 2018;9:934. 30210339 10.3389/fphar.2018.00934 PMC6121093

[104] Xia F Liu C Wan JB. Characterization of the cold and hot natures of raw and processed Rehmanniae Radix by integrated metabolomics and network pharmacology. Phytomedicine. 2020;74:153071. 31537418 10.1016/j.phymed.2019.153071

[105] Zhou L Xu JD Zhou SS. Integrating targeted glycomics and untargeted metabolomics to investigate the processing chemistry of herbal medicines, a case study on Rehmanniae Radix. J Chromatogr A. 2016;1472:74–87. 27771102 10.1016/j.chroma.2016.10.043

[106] Li M Wang X Han L. Integration of multicomponent characterization, untargeted metabolomics and mass spectrometry imaging to unveil the holistic chemical transformations and key markers associated with wine steaming of Ligustri Lucidi Fructus. J Chromatogr A. 2020;1624:461228. 32540070 10.1016/j.chroma.2020.461228

[107] Su X Wu Y Li Y. Effect of different post-harvest processing methods on the chemical constituents of notopterygium franchetii by an UHPLC-QTOF-MS-MS metabolomics approach. Molecules. 2019;24:3188. 31480764 10.3390/molecules24173188 PMC6749590

[108] Xia F Liu C Wan JB. Characterization of the cold and hot natures of raw and processed Rehmanniae Radix by integrated metabolomics and network pharmacology. Phytomedicine. 2020;74:153071. 31537418 10.1016/j.phymed.2019.153071

[109] Ding M Li Z Yu XA. A network pharmacology-integrated metabolomics strategy for clarifying the difference between effective compounds of raw and processed Farfarae flos by ultra high-performance liquid chromatography-quadrupole-time of flight mass spectrometry. J Pharm Biomed Anal. 2018;156:349–57. 29753281 10.1016/j.jpba.2018.05.003

[110] Dai Y Li Q Tong J. Quality marker identification based on standard decoction of differently processed materials of Ephedrae Herba. J Ethnopharmacol. 2019;237:47–54. 30898554 10.1016/j.jep.2019.03.025

[111] Zhang BX Qi XJ Cai Q. Metabolomic study of raw and bran-fried Atractylodis Rhizoma on rats with spleen deficiency. J Pharm Biomed Anal. 2020;182:112927. 32007825 10.1016/j.jpba.2019.112927

[112] Zhong LJ Hua YL Ji P. Evaluation of the anti-inflammatory effects of volatile oils from processed products of Angelica sinensis radix by GC-MS-based metabolomics. J Ethnopharmacol. 2016;191:195–205. 27292195 10.1016/j.jep.2016.06.027

[113] Yuan Z Zhong L Hua Y. Metabolomics study on promoting blood circulation and ameliorating blood stasis: investigating the mechanism of Angelica sinensis and its processed products. Biomed Chromatogr. 2019;33:e4457. 30520064 10.1002/bmc.4457

[114] Tao Y Huang S Yan J Li W Cai B. Integrated metallomic and metabolomic profiling of plasma and tissues provides deep insights into the protective effect of raw and salt-processed Achyranthes bidentata Blume extract in ovariectomia rats. J Ethnopharmacol. 2019;234:85–95. 30784959 10.1016/j.jep.2019.01.033

[115] Yan Y Du C Li Z. Comparing the antidiabetic effects and chemical profiles of raw and fermented Chinese Ge-Gen-Qin-Lian decoction by integrating untargeted metabolomics and targeted analysis. Chin Med. 2018;13:54. 30386417 10.1186/s13020-018-0208-7 PMC6204051

[116] Kong M Liu HH Wu J. Effects of sulfur-fumigation on the pharmacokinetics, metabolites and analgesic activity of Radix Paeoniae Alba. J Ethnopharmacol. 2018;212:95–105. 29080828 10.1016/j.jep.2017.10.023

[117] Han Y Li Y Wang Y Gao J Xia L Hong Y. Comparison of fresh, dried and stir-frying gingers in decoction with blood stasis syndrome in rats based on a GC-TOF/MS metabolomics approach. J Pharm Biomed Anal. 2016;129:339–49. 27454085 10.1016/j.jpba.2016.07.021

[118] Han L Wang P Wang Y. Rapid discovery of the potential toxic compounds in polygonum multiflorum by UHPLC/Q-Orbitrap-MS-based metabolomics and correlation analysis. Front Pharmacol. 2019;10:329. 31057397 10.3389/fphar.2019.00329 PMC6477936

[119] Zhang W Jiang H Yang J. A high-throughput metabolomics approach for the comprehensive differentiation of four Pulsatilla Adans herbs combined with a nontargeted bidirectional screen for rapid identification of triterpenoid saponins. Anal Bioanal Chem. 2019;411:2071–88. 30734858 10.1007/s00216-019-01631-6

[120] Zhang J Wang P Wei X. A metabolomics approach for authentication of Ophiocordyceps sinensis by liquid chromatography coupled with quadrupole time-of-flight mass spectrometry. Food Res Int. 2015;76(Pt 3):489–97. 28455029 10.1016/j.foodres.2015.07.025

